# A Ticking Time Bomb: A Case Report of Neutropenic Fever Secondary to Tick-Borne Illness

**DOI:** 10.7759/cureus.69585

**Published:** 2024-09-17

**Authors:** Danielle C Thor, Joann Y Ha, Yasemin Galiboglu, Kristine Wong, Cindy Hou

**Affiliations:** 1 Internal Medicine, Jefferson Health New Jersey, Stratford, USA; 2 Internal Medicine, Rowan-Virtua School of Osteopathic Medicine, Stratford, USA; 3 Infectious Diseases, Jefferson Health New Jersey, Stratford, USA

**Keywords:** babesia, babesiosis, borrelia, hematology, infectious disease, lyme disease, neutropenia, neutropenic fever, oncology, tick-borne illness

## Abstract

The advent of immunomodulatory therapies and their ever-expanding number of treatment indications necessitates the understanding of their associated complications. Neutropenic fever serves as an example of these complications often encountered in clinical practice. Although neutropenic fever can result from virtually any pathogen, episodes of the syndrome secondary to tick-borne illness remain relatively undocumented in the scientific literature. In the case presented, a 77-year-old female with a pertinent past medical history of smoldering IgG multiple myeloma on active immunosuppressive therapy presented with a first-time episode of neutropenic fever likely secondary to tick-borne illness. Through this report, attention is drawn to an additional source pathogen for neutropenic fever and its management, thus expanding upon clinician understanding of this all-too-common complication of immunosuppression.

## Introduction

Scientific advances in pharmacotherapy have championed a variety of immunogenic products, ranging from traditional chemotherapies to immunotherapies and cellular therapies, for exponentially increasing treatment indications. However, these expansive therapies are not without sequelae and often result in their recipients becoming immunocompromised. Approximately 6.6% of all adult patients in the United States are immunocompromised from all causes including both pharmacotherapy and other intrinsic immunodepleting pathology [1]. Many of these patients are subsequently at risk for relevant complications, such as neutropenic fever. Neutropenic fever is defined as a single oral temperature greater than or equal to 101°F (38.3°C) or a temperature greater than or equal to 100.4°F (38.0°C) sustained for at least one hour in the setting of an absolute neutrophil count (ANC) less than or equal to 1500 cells/microliter or 1.5 B/L [2]. Considering the majority of immunomodulating therapy is utilized in the oncological setting, mortality for neutropenic fever in pediatric cancers, solid tumors, and hematological malignancies have been established at 0.4-3.0%, 2.6-7.0%, and 7.4%, respectively [3].

Although virtually any infection in an immunocompromised person can result in a neutropenic fever, several pathogens are more often implicated in this presentation. Bacterial infections tend to be the most common culprit over viral or fungal sources, with gram-positive and gram-negative bacteria infection rates occurring in a 60:40 percent ratio [4]. However, despite these observations, most neutropenic fevers are classified as fevers of unknown origin, due to the lack of definitive microbiological findings [5]. Inpatient treatment regimens therefore consist initially of broad-spectrum antibiotics, such as cefepime or piperacillin/tazobactam with or without vancomycin, and are deescalated thereafter depending on whether a source organism is identified and/or individual and regional antibiotic sensitivity profiles [5]. 

Of the spectrum of causal agents of neutropenic fever, tick-borne illness is not typically observed, with virtually no prior reports available in the scientific literature. In the case presented below, the diagnosis and management of a first-time episode of neutropenic fever likely secondary to tick-borne illness is documented. In doing so, attention is provided to an additional potential cause of this condition to further guide clinicians in this increasingly common inpatient presentation.

## Case presentation

The patient is a 77-year-old female with a past medical history of smoldering IgG multiple myeloma, atrial fibrillation (status-post cardioversion four months prior to arrival), prior pulmonary embolism (on active anticoagulation therapy), hypertension, and pituitary microadenoma, who presented due to worsening fevers with chills over the past 24-48 hours prior to arrival. On initial examination, she stated that she experienced subjective fevers and chills over the past 48 hours prior to arrival and found her temperature to be elevated to 102.5°F approximately 24 hours prior to arrival. The patient proceeded to self-dose over-the-counter acetaminophen and “sleep it off.” Upon waking in the morning prior to her arrival, her self-measured temperature was elevated to 103.5°F. She contacted her oncology nurse navigator and followed her recommendations thereafter to seek further care at her local emergency department.

Of note is that the patient endorsed extensive travel and insect exposure histories. She noted that she had traveled through France for nine days approximately five weeks prior to arrival. She contracted a COVID-19 infection upon her arrival home from France four weeks prior to arrival and simultaneously noted an acute, right-sided inflammatory hearing loss from this infection with an incomplete recovery. She denied ear pain, headaches, dizziness, or any fluid drainage from the ears at any point in time since her hearing loss onset. In addition, the patient described extensive exposure to insects and/or tick-borne illnesses. She noted that her primary residence is located in a densely forested area and that she and her spouse have had multiple tick bites in their years living there. Because of this, her spouse has had several symptomatic Lyme disease infections, as well as a prior case of babesiosis. More recently, her dog was admitted to a veterinary hospital for successful management of an infected abscess with an associated pericardial effusion approximately one week prior to arrival. Canine abscess cultures were available at the time of the patient’s admission and were found to be growing anaplasmosis; the pericardial effusion was attributed to a clinically presumed concurrent Lyme disease infection. Lastly, the patient described a mechanical fall after tripping over a street curb approximately three days prior to arrival, resulting in associated lacerations and bruising noted on her initial exam. 

Over the past three years prior to her admission, the patient was receiving active treatment for her smoldering IgG multiple myeloma, which consisted of quadruple therapy (daratumumab, dexamethasone, bortezomib, and lenalidomide) until progression, an autologous stem cell transplant, and maintenance therapy with pomalidomide and daratumumab thereafter with subsequent dose reductions due to intermittent neutropenia. Approximately two weeks prior to admission, the patient was found to be neutropenic yet again by her outpatient oncologist, and her treatment regimen was reduced to pomalidomide monotherapy pending further cellular recovery. Furthermore, the patient endorsed no prior hospitalizations other than two planned admissions for her cardioversion four months prior and her autologous stem cell transplant two years prior to arrival. 

**Figure 1:**
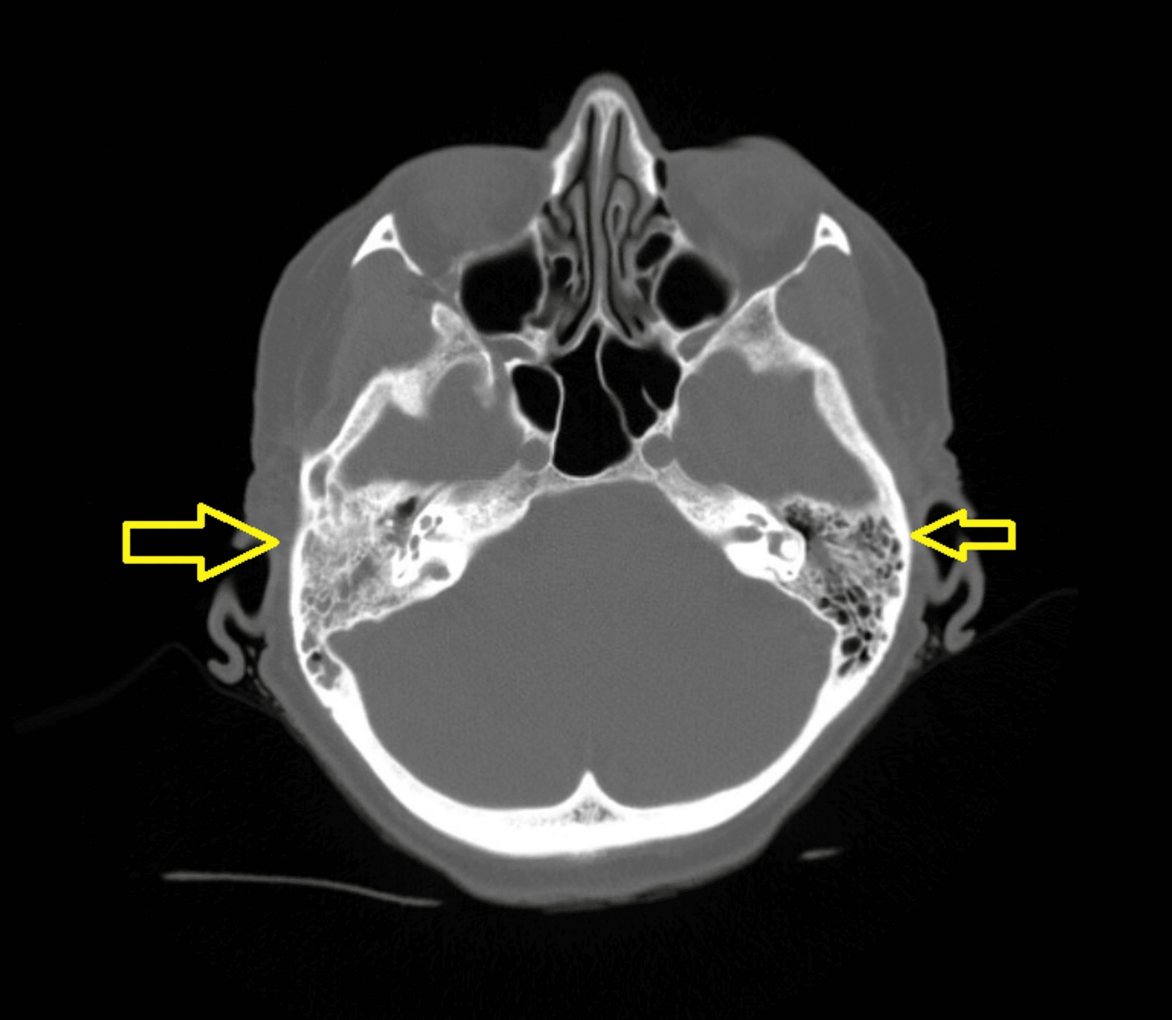
Findings from the patient’s computed tomography (CT) of the head indicating otomastoiditis, with a right-sided predominance as indicated by the larger arrow.

**Figure 2:**
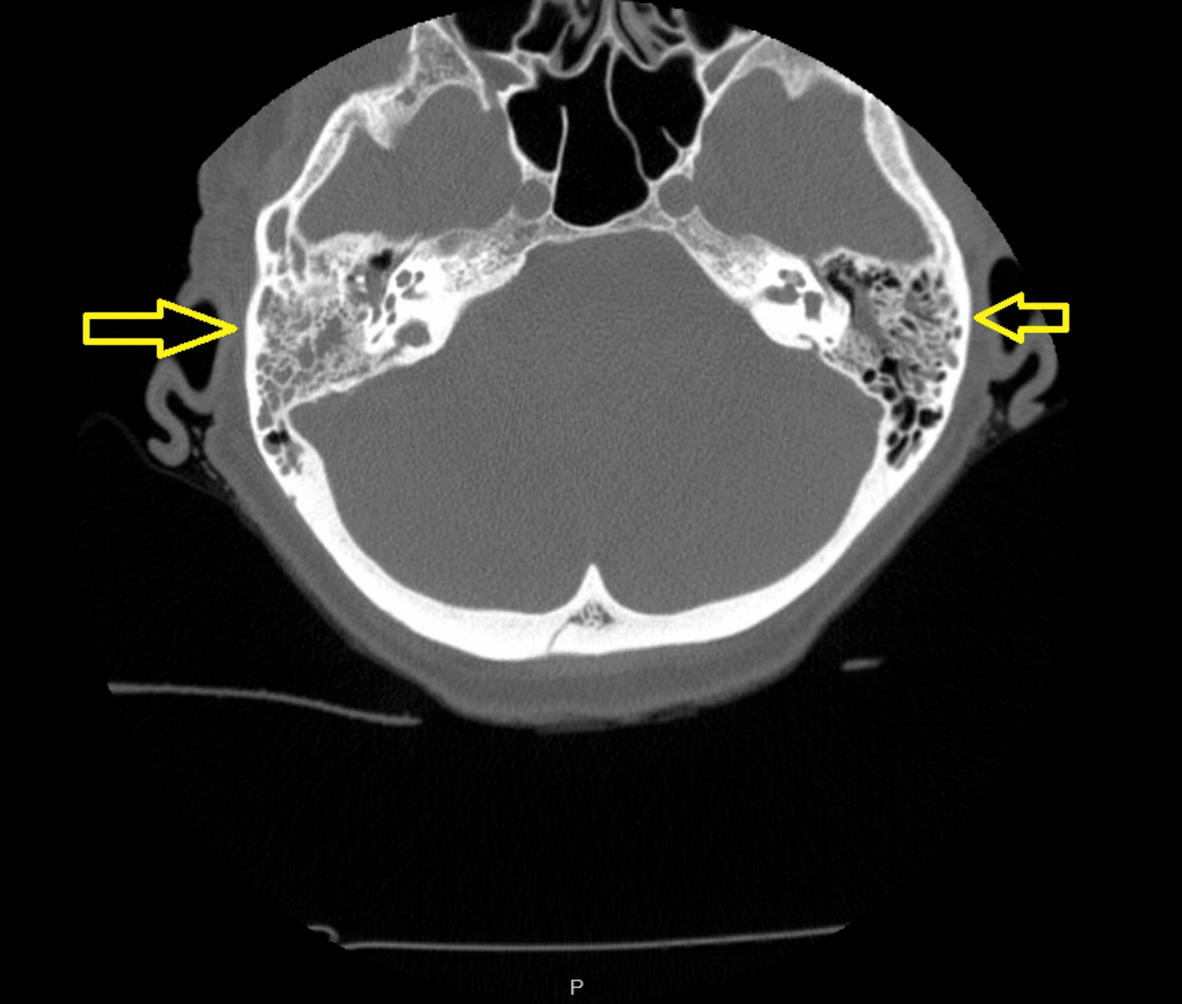
Findings from the patient’s computed tomography (CT) of the cervical spine indicating otomastoiditis, with a right-sided predominance as indicated by the larger arrow.

At the time of admission, the patient was found to be hypertensive with a maximum blood pressure of 176/70 and febrile with a maximum temperature of 102.3°F. Her initial laboratory workup was notable for a white blood cell (WBC) count of 2.6 B/L (normal: 3.7-10.5 B/L), an absolute neutrophil count (ANC) of 2.00 B/L (normal: 1.60-8.00 B/L), a hemoglobin (Hgb) of 11.7 g/dL (normal: 11.7-15.0 g/dL), and a platelet count (Plt) of 96 B/L (normal 150-400 B/L). Computed tomography (CT) of the chest, abdomen, and pelvis was obtained on admission but was negative for any abscess or other acute infectious findings. CT of the head (Figure 1) and cervical spine (Figure 2) was also obtained and was negative for acute traumatic findings, but notable for bilateral otomastoiditis with the right-sided findings more prominent than the left. The decision was made to hold all antineoplastic therapy, begin tailored intravenous antibiotic therapy with ceftriaxone and doxycycline due to the exposures noted above, and admit the patient to the general medicine service for further management of neutropenic fever likely secondary to tick-borne illness. (Of note, although the patient did not meet the technical criteria for neutropenic fever at the time of admission, her clinical presentation and eventual worsening of her neutropenia as later discussed provided multidisciplinary justification for treatment of her case as a neutropenic fever.)

The patient’s care was managed in conjunction with the on-service infectious disease physician, and his treatment regimen was broadened to cefepime and doxycycline to include all potential sources of neutropenic fever. An extensive neutropenic fever workup was collected at the time of admission and analyzed throughout the patient’s hospital course. The patient was found to have an unremarkable urinalysis and peripheral microbiology blood smear, as well as negative blood cultures, negative COVID-19/influenza screening tests, and a negative (1-3)-B-D-glucan fungal infection screen. A comprehensive tick-borne illness panel was collected, with the results provided in Table 1.

**Table 1:** Findings from the patient’s comprehensive tick-borne illness panel

Name	Result	Normal Range
Anaplasma phagocytophilum IgG	<1:64	<1:64
Anaplasma phagocytophilum IgM	<1:20	<1:20
Babesia microti IgG (blood)	1:128	<1:64
Babesia microti IgM (blood)	<1:20	<1:20
Ehrlichia chaffeensis IgG	<1:64	<1:64
Ehrlichia chaffeensis IgM	<1:20	<1:20
Rickettsia IgG	[Not detected]	[Not detected]
Rickettsia IgM	[Not detected]	[Not detected]
Borrelia burgdorferi (Lyme disease) antibody	Positive	Negative
Borrelia burgdorferi (Lyme disease) confirmatory IgG	Positive	Negative
Borrelia burgdorferi (Lyme disease) confirmatory IgM	Negative	Negative

**Figure 3:**
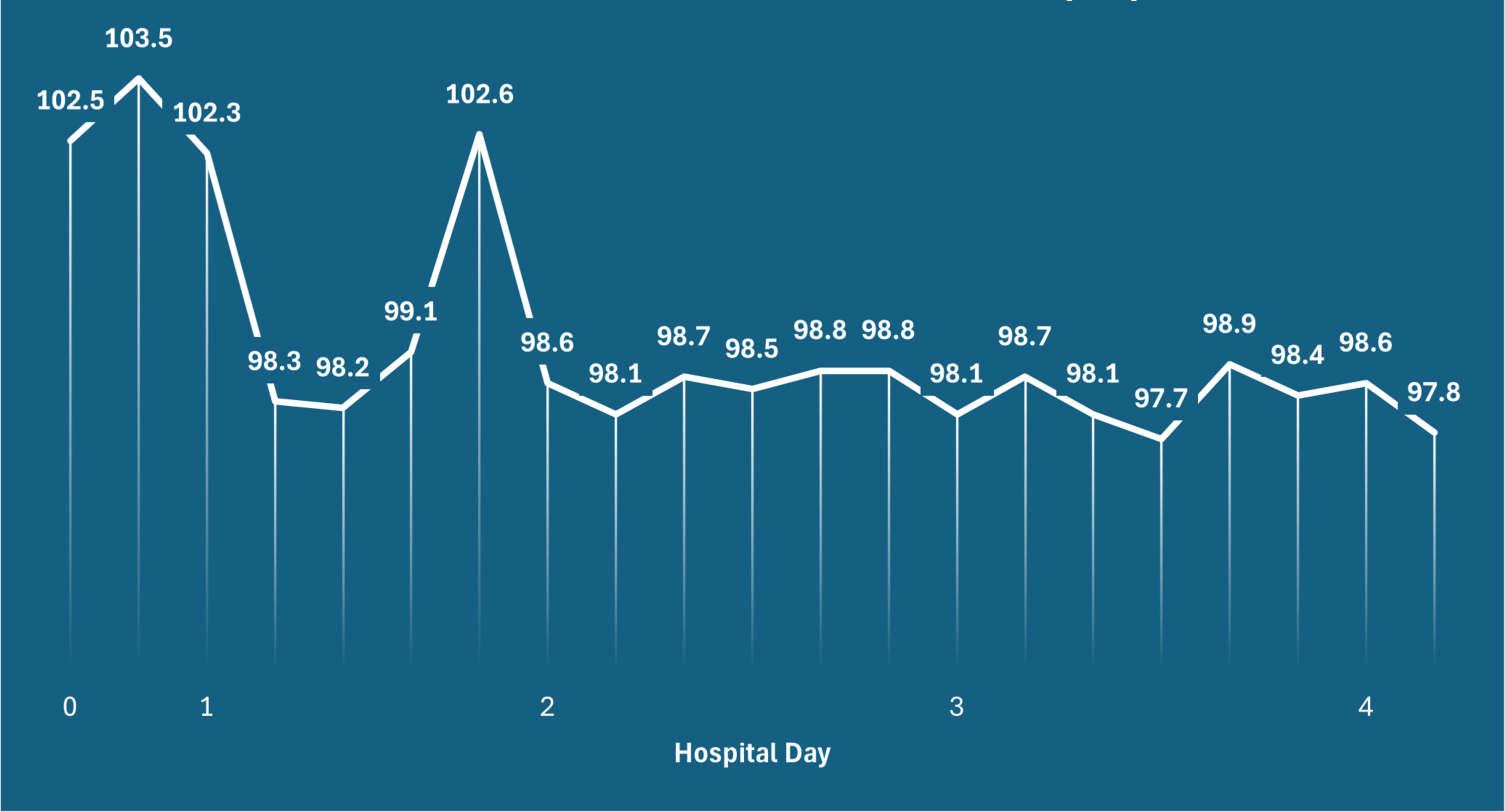
A graphical representation of the patient’s fever curve during her admission in degrees Fahrenheit.

**Figure 4:**
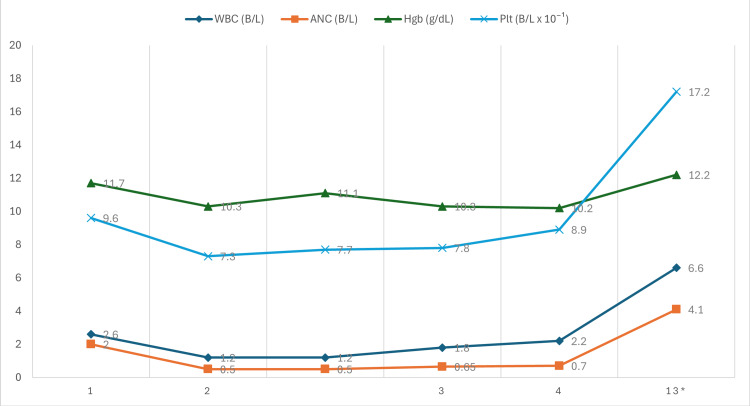
A graphical representation of the patient's white blood cell (WBC), absolute neutrophil count (ANC), hemoglobin (Hgb), and platelet (Plt) trends during her admission. *Day 13 represents her one-week post-hospitalization follow-up.

The patient’s hospital course was relatively uncomplicated, with a gradual resolution of her fevers (Figure 3), and a further decrease and subsequent rebound in her WBC, ANC, and Hgb levels (Figure 4). On this admission, she was also examined by the on-service otolaryngologist due to her previously reported hearing loss. This hearing loss was ultimately attributed to bilateral serous otitis media and serous mastoid effusions likely secondary to inflammatory changes following her recent COVID-19 infection. A shared decision was made among the patient, her primary medicine team, her infectious disease team, and her otolaryngology team to treat her with a combined antibiotic and steroid regimen to both cover her tick-borne illness while simultaneously preventing further hearing loss from inflammation secondary to her ear pathology. The patient was ultimately discharged with an oral antibiotic regimen of cefdinir and doxycycline, as well as an extended prednisone taper. Data from a one-week post-hospitalization follow-up with her oncologist were available and noted adequate resolution of her neutropenia, anemia, and thrombocytopenia (Figure 4).

## Discussion

As immunosuppressive therapies increase in their availability, continued development of the clinical understanding of neutropenic fever remains warranted. Neutropenic fever is one of the most common and serious adverse events of antineoplastic therapy, particularly among patients with hematological malignancies [5]. The incidence of all-cause tick-borne illness in the United States also continues to rise, secondary to accelerated tick population growth likely due to climate change [6-7]. As presented in this vignette, tick-borne illness was the likely source of this patient’s first episode of neutropenic fever in the setting of her active treatment of her smoldering IgG multiple myeloma. Her lack of significant prior hospital exposures and association of her ear pathology with post-viral changes instead of an acute infectious process further delineates tick-borne illness as a source pathogen in this case.

When diagnosing acute tick-borne illnesses, most blood-based assays rely on two-tier IgM and IgG assays [8]. Classic microbiology teachings note the presence of the “Maltese cross sign” on a peripheral blood smear as a diagnostic for babesiosis; however, this finding is only occasionally seen in practice [9]. Obvious increases in babesia serologies and a concurrent clinical picture provide a more realistic diagnosis. Although this patient was found to have positive IgG antibodies for *Babesia microti* and *Borrelia burgdorferi*, the two-tiered assay system should be interpreted more often than not as a true indicator of a prior, cleared infection. In addition, the unique pathology of multiple myeloma predisposes the patient to a range of immune dysfunctions that require more careful interpretation of antibody-based assays and a greater reliance on clinical judgment [10].

In this scenario, the patient’s pancytopenias documented by her outpatient oncologist and on admission were not anticipated to not be solely treatment-related given the design and dose reductions of her current multiple myeloma maintenance therapy. There is instead a possibility that her tick-borne illness began with a self-limited, physically asymptomatic babesiosis and the subsequent cell line reductions typically observed with this infection weeks prior to her admission [11]. Her physically symptomatic phase was more likely induced by a concurrent Lyme disease infection, as it is both the most common tick-borne illness and responded appropriately to doxycycline therapy [12].

When selecting treatment options for neutropenic fever, clinical judgment again remains prudent in antibiotic selection and duration. Although this patient endorsed no prior hospitalizations outside of two planned admissions months to years prior to her arrival, her overall burden of exposure to healthcare settings from her frequent oncologist visits led her clinicians to pursue broad-spectrum antibiotics beyond the coverages necessary to treat tick-borne illness alone. Her initial regimen of cefepime and doxycycline and transition regimen of cefdinir and doxycycline provided adequate coverage of the breadth of tick-borne illnesses, as well as continued treatment for any potentially bacterially mediated otitis media. The tailoring of her antibiotic regimen based on her likely exposures allowed for greater ease in the decision to concurrently treat her ear pathology with steroid therapy. 

A single-patient case report is inherently limited by its sample size and room for confounding variables. However, this patient’s unique medical, travel, and/or exposure histories provide an insightful picture of an additional complication for immunosuppressed persons throughout the Northeast Corridor of the continental United States and beyond. Ultimately, continued detailing of cases of neutropenic fever with clear sources of infection will help broaden the differential diagnoses of treating clinicians who may encounter these patients throughout the breadth of medical practice.

## Conclusions

This case report details an immunocompromised, adult female undergoing diagnosis and treatment of a first-time episode of neutropenic fever likely secondary to tick-borne illness. Through this report, a clinically relevant case is newly documented in the scientific literature to further advise clinicians in assembling an appropriately broad diagnosis for this significant complication in the oncological space and beyond.
